# Vitamin D supplementation in obese Sri Lankan children: a randomized controlled trial

**DOI:** 10.1186/s12887-020-02329-w

**Published:** 2020-09-05

**Authors:** D. B. D. L. Samaranayake, S. G. S. Adikaram, N. Atapattu, K. M. D. L. D. Kendaragama, J. T. N. Senevirathne, H. D. Jayasekera, V. P. Wickramasinghe

**Affiliations:** 1 Department of Community Medicine, Faculty of Medicine, University of Colombo, Colombo, Sri Lanka; 2 Colombo South Teaching Hospital, Kalubowila, Sri Lanka; 3 Lady Ridgeway Hospital, Colombo, Sri Lanka; 4 Department of Paediatrics, Faculty of Medicine, University of Colombo, Colombo, Sri Lanka

**Keywords:** Childhood obesity, Insulin resistance, Randomized controlled trial, Sri Lanka, Vitamin D deficiency

## Abstract

**Background:**

Micronutrient deficiencies are identified among obese individuals. Vitamin D deficiency (VDD) is prevalent in obese children, and is hypothesized to cause insulin resistance and metabolic abnormalities. This study aimed to determine the effect of vitamin D supplementation on obesity and related metabolic abnormalities among obese Sri Lankan children with VDD.

**Methods:**

A triple-blind randomized controlled trial was conducted among vitamin D deficient (< 20 ng/ml), obese children (*n* = 96), randomly allocated to three intervention arms - treatment arm receiving weekly vitamin D_2_ 50,000 IU; supplementation arm receiving 2500 IU weekly and control arm, receiving placebo. Anthropometry, percentage fat mass (%FM) and blood pressure were assessed and fasting blood glucose, fasting insulin, lipid profile, aspartate transaminase (ALT), alanine transaminase (AST), vitamin D, parathyroid hormone (PTH) and hs-CRP and OGTT with 2-h random blood glucose and insulin was performed at baseline and after 24 weeks of treatment. Ethics Review Committee of Faculty of Medicine, University of Colombo approved the protocol.

**Results:**

Waist circumference Z-score, %FM and serum calcium significantly improved across all three arms, ALT significantly improved in treatment and supplementation arms while, BMI Z-score, PTH and vitamin D significantly improved in the treatment arm. Biceps (*p* = 0.035) and subscapular (0.048) skin fold thickness, vitamin D (*p* = 0.004) and ALT (*p* = 0.012) significantly improved in the treatment arm.

**Conclusions:**

A strict dietary and physical activity regimen could improve some of the anthropometric, body composition and metabolic profiles, but high dose vitamin D, enhances those improvements. Therefore high dose vitamin D seems to potentiate management outcomes of obese children with vitamin D deficiency.

**Trial registration:**

The study was registered at the Sri Lanka Clinical Trials Registry (SLCTR/2015/017) on 12th September 2015 at https://slctr.lk/trials/slctr-2015-017.

## Background

The incidence of non-communicable diseases (NCD) is increasing more rapidly in developing countries than in industrialized countries. Obesity during childhood is a growing concern and the resulting socioeconomic and public health burden is enormous. Contrary to the popular belief that obese individuals are adequately nourished, micronutrient deficiencies have been identified to be prevalent among obese across all age groups worldwide.

Vitamin D is an essential nutrient that plays an important role in calcium homeostasis and it also plays a crucial role in insulin secretion and maintaining glucose homeostasis via its endocrine mechanisms [1–3]. As much as identifying the magnitude of the problem of vitamin D deficiency (VDD) among obese children, it is also important to identify the part it plays in insulin resistance among them. Obese individuals were noted to have VDD associated with other micronutrients in several studies, although the exact mechanism has not been revealed. Possible hypothesis for the lower 25-hydroxy vitamin D (25(OH)D) levels in obese children, includes decreased sun exposure due to sedentary lifestyle, poor quality diet and increased clearance of 25(OH)D due to being stored in adipose tissue [4].

Potential explanations for the association between low 25(OH)D level and impaired glucose tolerance have largely focused on direct effects of vitamin D on pancreatic β-cell insulin secretion. Vitamin D receptors and vitamin D-binding proteins are known to exist in pancreatic tissue, and calcium plays an important role in insulin secretion from β-cell [1, 5]. Other possible mechanism is the potential anti-inflammatory role of vitamin D. Since the immune-modulatory functions of vitamin D are incontestable, its deficiency in obese patients may coincide with enhanced systemic inflammation. In paediatric population, an association between low vitamin D level, and activation of pro-inflammatory, pro-diabetic and atherogenic pathways among the obese has been noted [6].

A study based on bi-directional genetic approach, has shown that a higher body mass index (BMI) leads to lower 25(OH)D. The effects of lower 25(OH)D in increasing BMI are likely to be small. Therefore population level interventions to reduce BMI are expected to decrease the prevalence of VDD [7]. Furthermore it might justify use of vitamin D in an attempt of preventing or treating obesity and metabolic syndrome.

Vitamin D supplementation in obese children has shown beneficial metabolic effects. It has shown to reduce serum triglyceride, insulin and insulin resistance levels, but no improvement in cholesterol, fasting blood sugar and blood pressure compared to a placebo group [8]. Furthermore, vitamin D supplementation has shown to improve BMI SD scores (BMI-SDS), alanine transaminase (ALT) and glycosylated haemoglobin (HbA1c) levels in type 2 diabetes mellitus compared to type 1 diabetes mellitus [9]. Studies in adults have also shown that reduction in weight has improved the vitamin D status and the improvement is proportionate to the weight loss [10]. Supplementation of vitamin D is available in two forms – vitamin D_2_ or ergocalciferol and vitamin D_3_ or cholecalciferol, both of which are prohormones, converted in to active compounds through a two-step hydroxylation process within the body. A recent systematic review and meta analysis shows that in bolus dose, vitamin D_3_ shows higher efficacy in raising blood level of 25(OH)D, however, no significant difference is seen with regard to daily supplementation [11].

Evidence on vitamin D status of Sri Lankan children is sparse. VDD using a higher cut off value (< 35 nmol/l) was seen in 26 and 25% of male and female children respectively in the Southern province of Sri Lanka [12]. A Similar study done more recently revealed VDD (< 20 nmol/l) in 29% of study population [13]. The prevalence of VDD (< 20 ng/ml) among obese Sri Lankan children was 75.2%, while insufficiency (20–30 ng/ml) was 21.3% [14].

In the light of ever increasing incidence of childhood obesity and associated higher prevalence of VDD, the current study was carried out to determine the effectiveness of vitamin D supplementation on obesity and related morbidities in children aged 5–15 years in an urban area of Sri Lanka.

## Methods

This was a randomized controlled trial with three parallel arms of equal size. Obese children aged 5–15 years attending the Obesity Clinic of Lady Ridgway Hospital, Colombo, who are identified to be having VDD (defined as 25 (OH) D < 20 ng/ml or < 50 nmol/L) [15] at a previously conducted cross-sectional study, were invited to participate in this prospective triple-blind placebo-controlled therapeutic and supplementary study [14]. Children who were suffering from any other chronic condition or were on long-term medication were excluded.

### Sampling

Sample size was calculated to determine a statistically significant difference in insulin resistance between the treatment group and control group. In a randomized controlled trial conducted by Belenchia et al. among obese (mean BMI-SDS + 2.53) adolescents with mean vitamin D concentration of 19.5 ng/ml, a statistically significant effect was shown on fasting insulin and HOMA-IR with treatment of vitamin D_3_ 4000 IU/d. According to their findings the mean HOMA-IR levels in the treatment and control group following 6 months of Vitamin D supplementation was 3.49 (SD = 1.84) and 5.0 (SD = 1.82) respectively [16]. Using this effect size and an α error of 5% and a β error of 20% (power of 80%) the sample size required was calculated as, 26 per treatment arm. Allowing for a non-response rate of 25%, a sample size of 35 was recruited for each treatment arm. Children who were identified as having VDD (25 (OH) D < 20 ng/ml) in the prevalence study were consecutively recruited until the required sample size was reached.

### Data collection method

#### Baseline assessment

Baseline assessment was conducted as part of the prevalence study, and data on socio-demograpics, anthropometry (height, weight, waist circumference, skinfold thickness), body composition using bio-impedance analysis using InBody 230® (InBody Inc., South Korea), puberty and biochemistry were assessed. For the biochemical assessment, blood was drawn after a 12-h overnight fast, for fasting blood glucose (FBG), lipid profile, aspartate transaminase (AST), alanine transaminase (ALT), high sensitive C-Reactive Protein (hs-CRP), serum insulin, 25(OH)D levels, serum Calcium, serum alkaline phosphatase (ALP) and serum parathyroid hormone (PTH) levels, 2-h random blood glucose (RBG) and insulin following oral glucose tolerance test (OGTT) [14].

#### Recruitment and random allocation

**Fig. 1:**
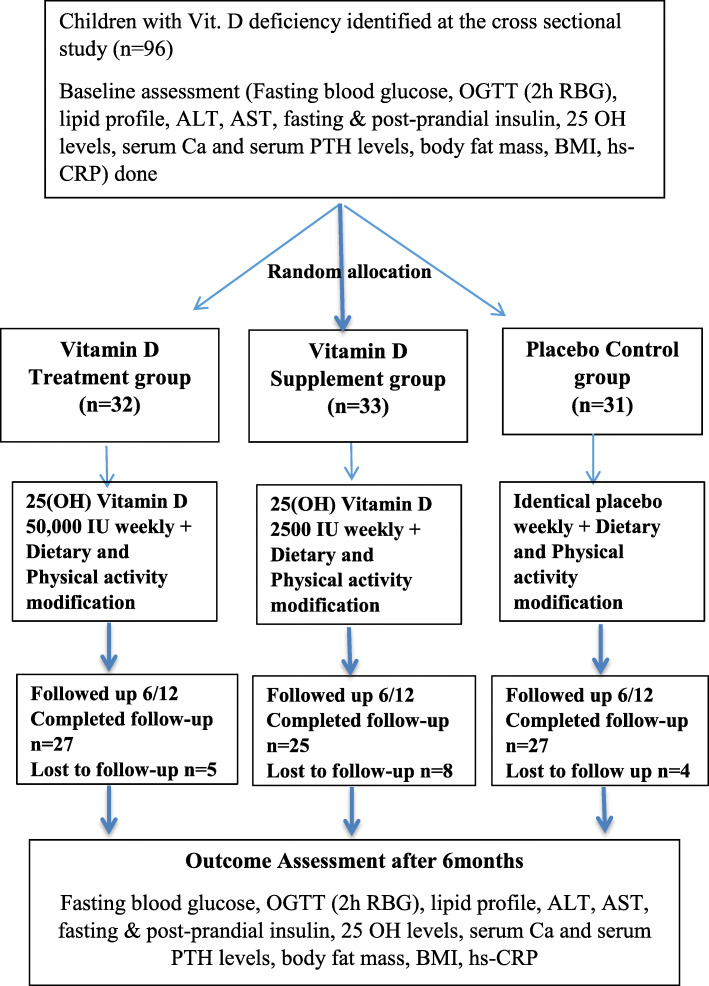
Participant flow from recruitment to completion of the trial in the three treatment arms – vitamin D treatment, vitamin D supplementation and placebo

The study was conducted at the Obesity Clinic of Lady Ridgway Hospital, Colombo. Study process was explained to the children recruited and informed, written consent was obtained from the parents/guardians. Once registered for the trial, the subjects were randomly allocated using a predetermined, computer-generated, concealed simple randomization sequence in to one of the three treatment arms - vitamin D in treatment doses, vitamin D in supplementation dose or placebo (Fig. 1). Principle investigator generated the randomization sequence and identical treatment packages were prepared for each participant according to the registration number. Allocation sequence of each participant was concealed from the recruiting officers who recruited and registered the participants, administered the treatment according to the registration numbers and conducted the data collection.

#### Intervention

Study subjects were followed up for 24 weeks with the respective treatments, structured dietary and physical activity interventions allocated to each treatment arm in the following manner:

- Treatment Group: Structured diet + Physical activity + Vitamin D_2_ treatment dose of 50,000IU weekly
- Supplementation Group: Structured diet + Physical activity + Vitamin D_2_ supplementation dose of 2500 IU weekly.
- Placebo Group: Structured diet + Physical activity + Placebo administered weekly

**Dietary advice** was given based on food based dietary guidelines produced by Ministry of Health [17]. Age-based portion size guide was given to parents and children and guidance was provided to them on the amount and the quality of the food they should be consuming.

**Physical activity advice** included instructions to engage in 60 min of daily physical activity, which would help to burn calories and improve fitness. Activities such as brisk walking, swimming or indoor or outdoor physical activity were recommended. Whenever they could not perform them as a single activity, were advised to carry out as short intensive sessions each lasting 15–20 min to obtain a cumulative outcome of 60 min per day.

**Treatment** comprised one of the following: Therapeutic dose of 50,000 IU of vitamin D_2_ (Ergocalciferol B.P., SGPharma®, Mumbai, India) weekly or a supplementary dose of vitamin D_2_ 2500 IU weekly, or an identical placebo. Of the two therapeutic forms of vitamin D, cholecalciferol is shown to be more effective than ergocalciferol in increasing 25(OH)D, when given in bolus doses. However, in daily supplementation doses, there is no significant difference between the two forms [11]. Since the present study used weekly doses, they were expected to behave in a manner similar to daily doses, hence similar efficacy of cholecalciferol and ergocalciferol was expected, and therefore, ergocalciferol was used as the therapy for both treatment and supplementation arms. All three treatments were administered in the form of a powder, and the participants were instructed to take is dissolved in a glass of milk and take on same day of the week at a similar time of the day.

#### Follow up and compliance

Participants were requested to keep a written record of their compliance with the treatment and physical activity recommendations. Telephone calls were given during the initial period to reinforce compliance. The participants were followed up monthly at the obesity clinic, Lady Ridgway Hospital. During the follow up visits the subjects were assessed for compliance to treatment, dietary advice and physical activity schedule and for adverse effects of the treatment. At each visit height, weight, waist circumference (WC), fat mass (FM) and blood pressure (BP) were recorded. Apart from these evaluations, after 12 weeks of intervention assessment of serum calcium (to screen for hypercalcemia) and urinary calcium to creatinine ratio (to screen for hypercalciuria) was done as a precautionary measure to detect any adverse response to vitamin D supplementation and no child showed adverse results.

#### Blinding

The study was designed as a triple-blinded trial. The study subjects were unaware of the treatment given. A blinded research officer carried out the assessment during each follow-up visit and at the end of 24 weeks. Statistical analysis of outcomes was conducted on a blinded data set identifying the three treatment arms using unrelated letters.

### Outcome assessment

Outcome assessment was conducted after 24 weeks of treatment. Primary outcome was the HOMA-IR while other metabolic (FBG, 2-h blood glucose following OGTT, fasting insulin, 2-h insulin, lipid profile, AST, ALT, hs-CRP, serum calcium, PTH) parameters, anthropometric (height, BMI and WC SD scores, SFT) parameters, body composition (percentage fat mass) parameters and systolic and diastolic blood pressure were defined as secondary outcomes. Anthropometric, body composition and metabolic parameters assessed at baseline were reassessed under the same conditions and according to the same standards specified. The laboratory assessments were performed in the endocrine laboratory of the Department of Obstetrics and Gynaecology, Faculty of Medicine, University of Colombo. The trial was conducted from October 2015 to February 2017 and the study ended after completing follow up of 24 weeks according to the protocol.

### Data analysis

Statistical analysis of data was conducted using SPSS-26 for windows. Intention-to-treat analysis, which maintains the prognostic balance generated from the original random treatment allocation and generates conservative estimates of treatment effect [18], was performed by substituting any missing values with latest available measurements. Baseline demographic, anthropometric, body composition and metabolic characteristics of the treatment, supplementary and control groups were compared using chi square test and One-way ANOVA test or relevant non-parametric tests. Between the three groups, the anthropometric, body composition and metabolic parameters at 24 weeks were compared using One-way ANOVA test or relevant non-parametric tests. Pre-post difference of the outcomes between the three groups was compared adjusting for their baseline values, using ANCOVA. Within the treatment and control groups the pre-post difference in the parameters were assessed using paired t-test or equivalent non-parametric tests.

### Ethics considerations

The Ethics Review Committee of Faculty of Medicine, University of Colombo approved the protocol (EC-16-030). Informed written consent was obtained from the parents of all participants and assent was obtained from the participants above the age of 12 years. The Clinical Trials committee of the National Medicine Regulatory Authority (NMRA) of Ministry of Health approved the protocol. The study was registered at the Sri Lanka Clinical Trials Registry accessible at https://slctr.lk (SLCTR/2015/017). CONSORT guidelines are adhered to, in reporting the methods and findings of the trial.

## Results

### Participant follow up and adherence to therapy

Final sample of *n* = 96, was randomized into vitamin D treatment arm (*n* = 32), vitamin D supplementation arm (*n* = 33) and placebo arm (*n* = 31). Complete follow up data were available for 79 subjects, 27 in treatment, 25 in supplementation and 27 in placebo arms. Intention to treat analysis was performed after substituting the missing values with the last available values. Analysis of the baseline characteristics of the subjects lost to follow up showed that they were not significantly different from those who completed the trial.

### Baseline characteristics

Table 1 shows the baseline characteristics compared between the three arms. Mean age was 9.8 years (SD 2.12) and 27 (28.1%) were girls. Age and gender distribution was similar in the three arms and there were no significant differences in mean systolic and diastolic blood pressure, anthropometry, body composition, serum vitamin D levels or other metabolic parameters between the three arms at baseline.

**Table 1:** Baseline characteristics of the sample randomized in to three intervention arms (*n* = 96)

Parameter		Supplementation (***n*** = 33) Mean (SD)	Treatment (***n*** = 32) Mean (SD)	Placebo (***n*** = 31) Mean (SD)
Age (years)		9.75 (2.26)	9.95 (2.02)	10.61 (1.83)
Gender*	Male	22 (66.7%)	22 (67.7%)	25 (80.6%)
	Female	11 (33.3%)	10 (32.3%)	6 (19.4%)
Ethnicity*^a^	Sinhala	23 (69.7%)	20 (64.5%)	23 (74.2%)
	Tamil	3 (9.1%)	4 (12.9%)	3 (10.5%)
	Muslim	7 (21.2%)	7 (19.4%)	5 (16.1%)
BMI SDS		2.83 (0.86)	2.72 (0.65)	2.66 (0.55)
Height SDS		0.64 (1.53)	0.66 (1.09)	0.50 (1.32)
Waist Circumference SDS		3.22 (0.72)	3.04 (0.62)	2.91 (0.56)
Percentage fat mass		43.31 (4.95)	41.89 (4.07)	41.94 (4.31)
Systolic BP SDS		−1.33 (0.82)	−1.29 (0.78)	− 1.41 (0.87)
Diastolic BP SDS		0.86 (0.77)	0.82 (0.64)	0.94 (0.68)
Vitamin D (ng/ml)		14.92 (3.92)	14.92 (3.04)	15.47 (2.78)
Parathyroid hormone (pg/ml)		36.41 (26.47)	37.24 (18.13)	34.19 (15.37)
Alkaline Phosphatase (IU/L)		262.79 (87.27)	243.0 (56.27)	270.29 (74.72)
Serum Calcium (mg/dl)		9.64 (0.46)	9.72 (0.48)	9.71 (0.58)
Fasting Blood glucose (mg/dl)		86.18 (8.62)	83.19 (5.28)	83.5 (6.17)
2-h OGTT (mg/dl)		119.25 (21.08)	112.48 (18.84)	116.63 (21.84)
Fasting Insulin (μIU/ml)		15.87 (8.96)	12.86 (8.58)	12.16 (8.47)
2-h Insulin (μIU/ml)		118.6 (85.3)	109.1 (88.2)	11.9 (88.0)
Total cholesterol (mg/dl)		198.27 (39.39)	183.94 (35.09)	176.98 (41.26)
Triglycerides (mg/dl)		108.36 (46.76)	103.53 (57.79)	129.16 (67.57)
HDL (mg/dl)		38.48 (7.28)	41.53 (10.22)	41.55 (9.59)
LDL (mg/dl)		138.11 (33.62)	123.11 (32.24)	115.63 (29.11)
AST (IU/l)		31.88 (10.29)	38.38 (31.85)	31.45 (15.38)
ALT (IU/l)		33.72 (21.39)	39.22 (51.37)	29.42 (27.73)
AST/ALT		1.1764 (0.68)	1.39 (0.71)	1.38 (0.77)
ALP (IU/L)		262.79 (87.27)	243.0 (56.27)	270.29 (74.72)
HS-CRP (mg/l)		4.41 (3.53)	3.83 (4.36)	3.562 (3.13)
Serum Ca (mg/dl)		9.64 (0.46)	9.72 (0.48)	9.71 (0.58)
HOMA IR		3.45 (2.12)	2.598 (1.79)	2.529 (1.81)

* Number and percentage are given ^a^One missing value was observed for ethnicity in treatment arm

*BMI* Body mass index, *WC* Waist circumference, *SDS* SD score, *SBP* Systolic Blood Pressure, *DBP* Diastolic Blood Pressure, *OGTT* Oral glucose tolerance test, *PTH* Parathyroid hormone, *FBS* Fasting Blood Sugar, *HDL* High-density Lipoprotein, *LDL* Low-density lipoprotein, *AST* Aspartate transaminase, *ALT* Alanine transaminase, *ALP* Alkaline phosphatase, *Hs-CRS* High sensitivity C reactive protein, *FM* Fat mass, *HOMA-IR* Insulin Resistance using homeostatic model

None of the parameters showed a statistically significant difference between the three intervention arms

### Outcome measures

Anthropometric and metabolic parameters at baseline and after 6 months were compared within the three treatment arms (Table 2). In the vitamin D treatment arm, BMI-SD score (2.72 to 2.52, *p* = 0.021), WC-SD score (3.04 to 2.75, *p* = 0.003), triceps SFT (27.36 to 24.88, *p* = 0.003) and %FM (41.89 to 38.75, *p* = 0.001) showed significant reductions from baseline to 6 months. Vitamin D levels (14.92 to 18.24, *p* = 0.003) increased while PTH (37.24 to 30.64, *p* = 0.006) and serum calcium (9.72 to 9.44, *p* = 0.013) levels decreased significantly. Fasting blood sugar levels, in contrast, showed a statistically significant increase (83.19 to 90.23, *p* = 0.000) from baseline to 6 months. Fasting insulin levels however, did not show any significant difference. A significant improvement was seen in mean HDL (41.53 to 49.25, *p* = 0.000) and ALT levels (39.22 to 21.66, *p* = 0.036). Non-significant improvements from baseline to 6 months were seen in biceps skin fold thickness, LDL levels, AST, AST/ALT ratio and ALP levels.

**Table 2:** Anthropometric and Metabolic Parameters at baseline and 6 months in the three intervention arms (*n* = 96)

Parameter	Supplementation (***n*** = 33)			Treatment (***n*** = 32)			Placebo (***n*** = 31)			Sig**.
	Baseline	6 months	Sig*.	Baseline	6 months	Sig*.	Baseline	6 months	Sig*.	
	Mean (SD)	Mean (SD)		Mean (SD)	Mean (SD)		Mean (SD)	Mean (SD)		
Height SDS	0.64 (1.53)	0.34 (1.38)	0.362	0.66 (1.09)	0.70 (1.07)	0.535	0.50 (1.32)	0.45 (1.29)	0.194	0.581
BMI SDS	2.83 (0.86)	2.70 (0.85)	0.142	2.72 (0.65)	2.52 (0.79)	**0.021**	2.66 (0.55)	2.57 (0.51)	0.073	0.297
WC SDS	3.22 (0.72)	2.89 (0.74)	**0.006**	3.04 (0.62)	2.75 (0.73)	**0.003**	2.9 (0.56)	2.69 (0.50)	**0.000**	0.369
Triceps SFT (cm)	26.93 (5.1)	26.04 (5.8)	0.415	27.36 (5.2)	24.88 (5.3)	**0.003**	27.05 (4.5)	25.48 (4.9)	0.117	0.548
Biceps SFT (cm)	20.65 (5.2)	20.26 (5.25)	0.725	19.69 (6.2)	17.75 (5.1)	0.065	19.56 (4.7)	20.1 (5.9)	0.547	0.121
Subscapular SFT (cm)	32.63 (8.6)	33.5 (8.6)	0.567	31.29 (7.3)	30.31 (7.3)	0.389	29.45 (5.3)	32.54 (6.6)	**0.006**	0.212
Suprailiac SFT (cm)	31.08 (6.6)	33.34 (6.4)	0.112	31.23 (5.9)	30.92 (6.1)	0.681	30.94 (5.9)	33.54 (5.4)	0.058	0.179
SBP SDS	−1.33 (0.82)	−1.26 (0.85)	0.685	−1.29 (0.78)	−1.236 (0.98)	0.786	−1.41 (0.87)	−1.16 (0.85)	0.256	0.863
DBP SDS	0.86 (0.77)	0.82 (.716)	0.834	0.82 (.645)	0.73 (.699)	0.562	0.94 (0.68)	0.97 (0.698)	0.842	0.447
Percentage FM	43.31 (4.95)	41.29 (5.46)	**0.000**	41.89 (4.068)	38.75 (5.7)	**0.001**	41.94 (4.31)	39.76 (5.43)	**0.008**	0.152
Vitamin D (ng/ml)	14.92 (3.92)	15.26 (3.675)	0.505	14.92 (3.04)	18.24 (5.77)	**0.003**	15.47 (2.78)	15.77 (3.43)	0.579	**0.020**
PTH (pg/ml)	36.41 (21.47)	33.94 (21.94)	0.325	37.24 (18.13)	30.64 (19.69)	**0.006**	34.19 (15.37)	29.23 (15.29)	0.137	0.648
FBS (mg/dl)	86.18 (8.62)	88.00 (7.79)	0.291	83.19 (5.28)	90.23 (8.82)	**0.000**	83.50 (6.17)	92.90 (11.15)	**0.000**	0.125
OGTT (mg/dl)	119.25 (21.08)	119.91 (17.79)	0.861	112.48 (18.84)	117.77 (25.43)	0.240	116.63 (21.81)	123.23 (19.48)	0.052	0.565
Fasting Insulin (μIU/ml)	15.87 (8.96)	14.09 (7.97)	0.198	12.86 (8.58)	15.19 (17.76)	0.406	12.16 (8.47)	13.312 (6.80)	0.364	0.854
2-h Insulin (μIU/ml)	118.60 (85.32)	115.57 (97.49)	0.847	109.096 (88.2)	94.89 (91.56)	0.496	111.947 (87.97)	94.70 (74.76)	0.092	0.581
Total cholesterol (mg/dl)	198.27 (39.39))	201.64 (43.37)	0.438	183.94 (35.09)	186.09 (38.59)	0.656	176.98 (41.26)	194.06 (32.64)	**0.034**	0.398
Triglycerides (mg/dl)	108.36 (46.76)	120.30 (53.79)	0.176	103.53 (57.79)	106.13 (61.20)	0.728	129.16 (67.57)	131.16 (74.84)	0.844	0.299
HDL (mg/dl)	38.48 (7.28)	45.61 (12.1)	**0.005**	41.53 (10.22)	49.25 (15.17)	**0.000**	41.55 (9.59)	48.65 (12.06)	**0.000**	0.392
LDL (mg/dl)	138.11 (33.62)	132.77 (39.81)	0.176	123.11 (32.24)	115.64 (33.67)	0.075	115.63 (29.11)	117.70 (30.34)	0.658	0.134
AST (IU/l)	31.88 (10.29)	29.24 (8.25)	0.075	38.38 (31.85)	31.19 (13.28)	0.075	31.45 (15.38)	31.71 (13.81)	0.883	0.706
ALT (IU/l)	33.72 (21.39)	25.97 (9.40)	**0.035**	39.22 (51.37)	21.66 (9.47)	**0.036**	29.42 (27.73)	26.94 (14.27)	0.543	0.142
AST/ALT	1.1764 (0.68)	1.1957 (0.34)	0.861	1.39 (0.71)	1.55 (0.59)	0.195	1.38 (.77)	1.283 (0.38)	0.499	**0.011**
ALP (IU/L)	262.79 (87.27)	240.52 (66.28)	**0.008**	243.0 (56.27)	231.0 (58.99)	0.099	270.29 (74.72)	240.39 (70.61)	**0.009**	0.796
HS-CRP (mg/l)	4.41 (3.53)	4.22 (3.7)	0.687	3.83 (4.36)	3.30 (4.26)	0.426	3.562 (3.13)	4.23 (5.09)	0.483	0.750
Serum Ca (mg/dl)	9.64 (0.46)	9.44 (0.53)	**0.034**	9.72 (0.48)	9.44 (0.51)	**0.013**	9.71 (0.58)	9.36 (0.61)	**0.013**	0.819
HOMA IR	3.45 (2.12)	3.099 (1.8838)	0.264	2.598 (1.79)	3.56 (4.96)	0.242	2.529 (1.81)	3.05 (1.66)	0.107	0.819

*Significance for the mean difference from baseline to 6 months – Paired samples T test **Significance between groups mean differences at 6 months – One way ANOVA test

*BMI* Body mass index, *WC* Waist circumference, *SDS* SD score, *SBP* Systolic Blood Pressure, *DBP* Diastolic Blood Pressure, *OGTT* Oral glucose tolerance test, *PTH* Parathyroid hormone, *FBS* Fasting Blood Sugar, *HDL* High-density Lipoprotein, *LDL* Low-density lipoprotein, *AST* Aspartate transaminase, *ALT* Alanine transaminase, *ALP* Alkaline phosphatase, *Hs-CRS* High sensitivity C reactive protein, *FM* Fat mass, *HOMA-IR* Insulin Resistance using homeostatic model

In the supplementation arm, mean WC-SD score (3.22 to 2.89, *p* = 0.006) and mean %FM (43.31 to 41.29, *p* = 0.000) showed significant reductions from baseline to 6 months. Mean HDL levels (38.48 to 45.61, *p* = 0.005) increased significantly while mean ALT (33.72 to 25.97, *p* = 0.035), ALP (262.8 to 240.5, 0.008) and serum calcium (9.64 to 9.44, *p* = 0.034) levels showed a significant reduction.

In the placebo-treated arm, statistically significant improvements from baseline to 6 months were seen in WC-SD score (2.90 to 2.69, *p* = 0.000), %FM (41.94 to 39.76, *p* = 0.008), HDL (41.55 to 48.65, *p* = 0.000), ALP (270.3 to 240.4, *p* = 0.008) and serum calcium (9.71 to 9.36, *p* = 0.013) levels. Subscapular SFT (29.45 to 32.54, *p* = 0.006) and FBG (83.5 to 92.9, *p* = 0.000) levels significantly deteriorated in the placebo arm during this period, while there was no significant change in the fasting insulin level.

Baseline to 6 months difference in the anthropometric and metabolic parameters adjusted for the baseline levels, were compared between the three arms (Table 3). Statistically significant differences were seen in the baseline to 6 months difference in vitamin D levels, biceps and subscapular SFT and ALT levels between the three treatment arms. Baseline to 6 months increase in vitamin D levels in the treatment arm (3.33 ng/ml) was significantly (*p* = 0.004) higher than that of the supplementation (0.28 ng/ml) and placebo (0.46 ng/ml) arms. Reduction in biceps SFT in the treatment arm (− 2.09) was significantly (*p* = 0.035) larger than the change in the supplementation arm (0.75). Similarly, the reduction in subscapular SFT in treatment arm (− 1.54) was significantly (*p* = 0.048) larger than the change in the placebo arm (2.38). ALT levels in the treatment arm (− 9.74) showed a significantly (*p* = 0.012) larger reduction compared to that of the placebo arm (− 2.77). Baseline to 6 months reductions of relatively larger magnitude in the treatment arm compared to supplementation and placebo arms were seen in BMI-SD score, triceps and suprailiac SFT, WC-SD score, percentage fat mass, serum parathyroid hormone, 2-h insulin, LDL cholesterol, serum AST, AST/ALT ratio and HOMA-IR. However, these differences were not statistically significant.

**Table 3:** Difference in anthropometric and Metabolic parameters from baseline to six months in the three intervention arms after adjusting to baseline levels (*n* = 96)

Parameter	Supplementation (***n*** = 33)		Treatment (***n*** = 32)		Placebo (***n*** = 31)		Sig.*
	Mean Difference	SE	Mean Difference	SE	Mean Difference	SE	
Height SDS	0.116	0.049	0.016	0.049	− 0.052	0.049	0.054
BMI SDS	−0.062	0.054	−0.135	0.056	−0.091	0.055	0.650
Triceps SFT (cm)	−0.437	0.776	−2.891	0.801	−1.520	0.801	0.095
Biceps SFT (cm)	0.752	0.780	− 2.091	0.780	−0.087	0.806	**0.035**
Subscapular SFT (cm)	1.384	1.101	−1.535	1.148	2.376	1.160	**0.048**
Supra-iliac SFT (cm)	2.256	0.968	−0.213	0.999	2.499	1.015	0.110
WC SDS	−0.222	0.072	−0.325	0.070	−0.230	0.069	0.521
SBP SDS	0.075	0.158	0.109	0.161	0.192	0.161	0.868
DBP SDS	−0.047	0.120	− 0.181	0.124	0.118	0.124	0.240
% FM	−1.639	0.533	−2.536	0.538	−1.807	0.538	0.458
Vitamin D (ng/ml)	0.282	0.696	3.331	0.718	0.463	0.719	**0.004**
PTH (pg/ml)	−1.004	2.224	−6.370	2.267	−2.396	2.311	0.224
FBG (mg/dl)	2.598	1.319	5.784	1.403	6.841	1.417	0.078
OGTT (mg/dl)	3.593	3.343	4.576	3.290	8.328	3.336	0.572
Fasting Insulin (μIU/ml)	−0.609	1.193	−0.602	1.200	0.551	1.181	0.729
2-h Insulin (μIU/ml)	−1.996	12.872	−25.056	13.092	−17.320	13.09	0.444
Total cholesterol (mg/dl)	5.223	4.731	1.662	4.827	10.138	4.936	0.470
Triglycerides (mg/dl)	10.970	7.952	0.514	8.234	10.369	8.416	0.601
HDL (mg/dl)	5.285	1.594	8.082	1.610	7.171	1.607	0.460
LDL (mg/dl)	−3.728	4.091	−7.838	3.999	0.340	4.078	0.361
AST (IU/L)	−2.832	1.315	−3.481	1.381	−0.133	1.358	0.188
ALT (IU/L)	−4.732	1.602	−9.740	1.650	−2.772	1.696	**0.012**
AST/ALT	−0.049	0.063	0.171	0.065	0.015	0.065	0.052
ALP (IU/L)	−20.688	6.740	−13.128	7.113	−21.082	7.080	0.672
HS-CRP (mg/L)	−0.184	0.433	0.239	0.437	0.659	0.445	0.408
Serum Ca (mg/dl)	−0.231	0.092	−0.272	0.095	−0.337	0.095	0.724
HOMA IR	−0.051	0.245	−0.209	0.255	0.384	0.246	0.220

*BMI* Body mass index, *WC* Waist circumference, *SDS* SD score, *SBP* Systolic Blood Pressure, *DBP* Diastolic Blood Pressure, *OGTT* Oral glucose tolerance test, *PTH* Parathyroid hormone, *FBS* Fasting Blood Sugar, *HDL* High-density Lipoprotein, *LDL* Low-density lipoprotein, *AST* Aspartate transaminase, *ALT* Alanine transaminase, *ALP* Alkaline phosphatase, *Hs-CRS* High sensitivity C reactive protein, *FM* Fat mass, *HOMA-IR* Insulin Resistance using homeostatic model, *SFT* Skin fold thickness

*Significance tested using ANCOVA adjusting for the baseline levels of each parameter

### Adverse events and compliance to treatment

No adverse events were reported in the study population. Urinary calcium to creatinine ratio remained below 0.14 in all the participants. Compliance was found to be satisfactory in all the subjects who completed the trial.

## Discussion

Vitamin D deficiency (VDD) and obesity are two global epidemics that are rising in the paediatric population with these two converging on each other where obesity has shown to a be strong risk factor for VDD and it emphasizes the need for more research to clarify the role of VDD on obesity and its related metabolic risks [19]. Dietary and life style changes could prevent VDD in obese children. However, vitamin D supplementation is recommended for those with inadequate intake, for those with deficient vitamin D levels or for individuals who are symptomatic of VDD. Doses are based on whether VDD is being prevented or treated [19].

The 2014 American Association of Paediatricians (AAP) clinical guidelines on bone health promotion recommend supplementation for obese children with inadequate vitamin D intake or VDD [20]. Screening for serum 25(OH)D may not be necessary as supplementation is more cost-effective than screening in the presence of clinical manifestations of hypocalcaemia, especially in regions where VDD is high and also resources limited [21]. Both the AAP and Endocrine Society of America recommend children with VDD to be supplemented with either 2000 IU/day or 50,000 IU/week of vitamin D_2_ or vitamin D_3_, at least for 6 weeks, followed by a maintenance dose of 600–1000 IU/day [20, 22]. However, obese children may need higher doses. Ideally serum levels should be assessed annually [21] and doses adjusted accordingly [22].

Recent evidence reports VDD showing significant associations with insulin resistance, metabolic syndrome and related cardio-metabolic abnormalities [6, 23, 24]. Vitamin D has an important role in glucose and insulin metabolism [25]. Association between serum vitamin D levels and insulin resistance is seen even in children, and vitamin D supplementation is proposed for children and adolescents to prevent insulin resistance, despite lack of consensus on the dose [26]. However, evidence lacks as to whether vitamin D supplementation improves insulin sensitivity and reduces metabolic risk in children and adolescents [8].

In the current study the metabolic markers related to insulin resistance did not show any significant improvement in the vitamin D treatment or supplementation groups compared to the control. Similarly, the expected improvement was not seen in FBG and 2-h blood glucose following OGTT. This lack of improvement could be due to the low dose of Vitamin D that was used in our study for treatment as well as supplementation. An Iranian study involving 10–16 year old obese children with metabolic syndrome were supplemented with 300,000 IU of oral vitamin D and observed a significant reduction in fasting insulin and HOMA-IR, although a significant reduction in FBG was not observed [8]. A study done in Australia involving overweight adults showed that there was no improvement in insulin secretion or insulin sensitivity at a dose which was higher than what we used [27]. In another study carried out in Turkey involving 10–18 year old obese children did not show significant improvement in blood sugars, insulin or HOMA-IR after supplementing with vitamin D 4000 IU/day for 3 months [28]. This lack of improvement in insulin resistance and glycaemic parameters with vitamin D supplementation in normoglycaemic subjects is highlighted in a systematic review and meta analysis of 15 trials, as well [29].

Results of the baseline assessment of this study clearly showed that obese children with VDD have associated changes such as increased levels of PTH and ALP while maintaining normal serum calcium levels. Treatment with vitamin D improved PTH and ALP levels and the reduction of calcium levels than baseline would be due to the removal of the PTH effect on bone resorption. Reduction in calcium and ALP level and non-significant reduction in PTH level was seen in the supplementation group as well supporting the same mechanism. However, the placebo group also has shown some improvement in these parameters, which could be due to reduction in the FM (as denoted by reduced WC and %FM) thus releasing more vitamin D to the circulation and bringing about its effects. Therefore, this study shows that vitamin D treatment is beneficial for obese children over and above simple supplementation or dietary and physical activity interventions. There was no significant difference in the vitamin D levels between supplementation and placebo arms at 6 months, hence supplementation with ergocalciferol low doses (2500 IU/week) seems to be ineffective in significantly improving vitamin D levels in obese children with VDD. Most available evidence on efficacy of vitamin D treatment and supplementation is on high doses well above 600 IU/d and there is limited evidence on low dose supplementation [11], therefore, the supplementation dose of 2500 IU/weekly of ergocalciferol used in the current study is likely to be inadequate.

Interestingly, our study showed significant improvement in ALT levels following treatment or supplementation with vitamin D, and this improvement in the treatment group was significantly higher than the other two. Pre-post difference in the AST/ALT ratio also was significantly higher in the treatment group compared to the other two. Improvement in hepatic markers is an important aspect in the management of obese children and there is limited evidence on the effect of vitamin D supplementation on hepatic markers. Therefore, the positive effect reported in the current study adds important evidence especially in a background of increasing prevalence of non-alcoholic fatty liver disease. There was no significant change shown in the liver enzymes in the Turkish study [28], and a recent review also highlights the need for further evidence to support this effect of vitamin D supplementation [30].

According to our findings, HDL showed a statistically significant increase in all three groups. Other fractions of lipid profile did not show any improvement while the total cholesterol levels in the placebo arm significantly deteriorated from the baseline value. Except for triglyceride levels none of the other components showed any improvement in Iranian children [8]. Total and LDL cholesterol showed a significant reduction in Turkish children who were supplemented with vitamin D, but there was no change in HDL or triglyceride levels [28]. Evidence on the effect of vitamin D on the lipid profile has been inconclusive as highlighted by a review of 19 randomized controlled trials [31]. In this background, our findings on the improvement of HDL denotes that diet and physical activity play a major role in lipid control, as much as vitamin D supplementation.

In the vitamin D treatment arm, BMI, WC, SFT and %FM improved significantly, while some of these parameters improved in the supplementation and placebo arms as well. Improvement in the biceps and subscapular SFT was significantly higher in the treatment arm. These findings show that vitamin D treatment has superiority over supplementation and placebo. However, diet and physical activity still play a part in improving the body composition of obese children. Overall, according to our findings vitamin D appears to play a part in insulin function and also in improving liver functions, which is likely to be mediated via insulin. This mechanism is quite plausible since non-alcoholic steatosis is considered as the hepatic component of metabolic syndrome. The current study was conducted at the Lady Ridgway hospital, which is the largest tertiary care children’s hospital in the country, and the Obesity Clinic is attended by a majority of children residing in the Western Province belonging to all ethnic groups. Hence the findings of the current study can be generalized to urban children of Sri Lanka.

### Limitations

The low response seen in most outcomes in the current study could be due the dose that we have used being inadequate, which is evident by the fact that even in the therapeutic group the mean vitamin D levels did not rise above the deficiency cut-off value of 20 ng/ml. Our sample size, although calculated for the primary outcome, may not have been adequate for the multiple secondary outcomes.

## Conclusions

This study shows that treatment with vitamin D_2_ 50,000 IU weekly in combination with diet and physical activity helps to improve insulin function and liver derangements as well as anthropometric and body composition parameters in obese Sri Lankan children with VDD, while minimal beneficial effects were seen in those supplemented with vitamin D_2_ 2500 IU weekly. As VDD prevalence is high in obese Sri Lankan children, it could be used as an adjunct therapy in the management. More research is needed using higher and different dosage regimens to identify the optimum regimen of vitamin D.

## Data Availability

The datasets used and/or analysed during the current study are available from the corresponding author on reasonable request.

## Abbreviations

AAP: American Academy of Paediatrics
ALP: Alkaline phosphatase
ALT: Alanine aminotransferase
AST: Aspartate aminotransferase
BMI: Body mass index
FBG: Fasting blood glucose
FM: Fat mass
HbA1c: Glycosilated haemoglobin
HDL: High density lipoprotein
HOMA-IR: Homeostatic Model Assessment of Insulin Resistance
hs-CRP: High sensitivity C reactive protein
IDF: International Diabetes Federation
LDL: Low density lipoprotein
NCD: Non Communicable Disease
OGTT: Oral glucose tolerance test
PTH: Parathyroid hormone
RBG: Random blood glucose
SDS: Standard deviation score
SFT: Skin fold thickness
SPSS: Statistical Package for Social Sciences
VDD: Vitamin D deficiency
WC: Waist circumference
